# Recent advances in *in silico* design and characterization of superalkali-based materials and their potential applications: A review

**DOI:** 10.3389/fchem.2022.1019166

**Published:** 2022-11-07

**Authors:** Sarvesh Kumar Pandey, Elangannan Arunan, Ratnesh Das, Atish Roy, Arunesh Kumar Mishra

**Affiliations:** ^1^ Department of Inorganic and Physical Chemistry, Indian Institute of Science Bengaluru, Bengaluru, Karnataka, India; ^2^ Department of Chemistry, Dr. Harisingh Gour University (A Central University), Sagar, Madhya Pradesh, India

**Keywords:** binding energy (BE), HOMO-LUMO (highest occupied molecular orbital-lowest unoccupied molecular orbital), ionization potential (IP), NLO (nonlinear optical), superalkali

## Abstract

In the advancement of novel materials, chemistry plays a vital role in developing the realm where we survive. Superalkalis are a group of clusters/molecules having lower ionization potentials (IPs) than that of the cesium atom (3.89 eV) and thus, show excellent reducing properties. However, the chemical industry and material science both heavily rely on such reducing substances; an *in silico* approach-based design and characterization of superalkalis have been the focus of ongoing studies in this area along with their potential applications. However, although superalkalis have been substantially sophisticated materials over the past couple of decades, there is still room for enumeration of the recent progress going on in various interesting species using computational experiments. In this review, the recent developments in designing/modeling and characterization (theoretically) of a variety of superalkali-based materials have been summarized along with their potential applications. Theoretically acquired properties of some novel superalkali cations (Li_3_^+^) and C_6_Li_6_ species, etc. for capturing and storing CO_2_/N_2_ molecules have been unveiled in this report. Additionally, this report unravels the first-order polarizability-based nonlinear optical (NLO) response features of numerous computationally designed novel superalkali-based materials, for instance, fullerene-like mixed-superalkali-doped B_12_N_12_ and B_12_P_12_ nanoclusters with good UV transparency and mixed-valent superalkali-based CaN_3_Ca (a high-sensitivity alkali-earth-based aromatic multi-state NLO molecular switch, and lead-founded halide perovskites designed by incorporating superalkalis, supersalts, and so on) which can indeed be used as a new kind of electronic nanodevice used in designing hi-tech NLO materials. Understanding the mere interactions of alkalides in the gas and liquid phases and the potential to influence how such systems can be extended and applied in the future are also highlighted in this survey. In addition to offering an overview of this research area, it is expected that this review will also provide new insights into the possibility of expanding both the experimental synthesis and the practical use of superalkalis and their related species. Superalkalis present the intriguing possibility of acting as cutting-edge construction blocks of nanomaterials with highly modifiable features that may be utilized for a wide-ranging prospective application.

## Introduction

Superalkalis are one distinct class of superatoms that consist of characteristics similar to those of alkali metals but have a substantially lower ionization potential (IP) than them (Wang et al., 1999). As a result, due to their low IPs, superalkalis quickly lose their valence electrons (Gutsev and Boldyrev, 1982; Khanna and Jena, 1995, Khanna and Jena, 2011). Gutsev and Boldyrev were the first to make use of the term “superalkali” in 1982 to refer to the distinct electronic structures of numerous radical forms of lithium and sodium-related species (Gutsev and Boldyrev, 1981). Recent research has proven that superalkalis are the superior supplier of extra electrons for creating novel materials with high initial hyperpolarizabilities than other materials that drop electrons rapidly from their outermost shell (Wang et al., 2012; Sun et al., 2014b, Sun et al., 2014a, Sun et al., 2016a). These species can give one electron to other molecules and survive as cations due to their IPs being lower than those of the alkali metal atoms (5.39–3.89 eV) in the periodic table of all elements (Lias et al., 1988) which means that they are more reactive even though they do not always resemble other elements (Wang et al., 2007). Researchers are constantly executing appropriate research work on the designing and characterization (experimentally or theoretically) of the superalkalis along with their potential applications. Nowadays, scientists have put significant effort into creating and describing diverse comical superalkali-based species.

The standard formula for superalkalis is M_k+1_L, where k is 1 for L (F, Cl, Br, and I) and 2 for L (O) atoms; Li_n_X (Velickovic et al., 2006; Velickovic et al., 2007; Velickovic et al., 2012) extended to K_2_X (X = F, Cl, Br, and I) (Velickovic et al., 2011) and Li_3_O (Zintl and Morawietz, 1938; Kudo et al., 1978; Wu et al., 1979) are two instances that come up frequently in this series. Li_2_F has been the subject of extensive theoretical (Gutsev and Boldyrev, 1982; Honea et al., 1989, Honea et al., 1993; Rehm et al., 1992; Koput, 2008; Wright et al., 2009) and experimental (Yokoyama et al., 2000; Neskovic et al., 2003; Fernandez-Lima et al., 2009) research. Well-known superalkalis, M_3_O (M = Li, Na, and K), have a greater propensity than their equivalent alkali atoms to lose an outer electron (Gutsev and Boldyrev, 1987; Rehm et al., 1992). Comprehensive experiments on the superalkalis having one nonmetal acting as the central atom (B, N, and O) connected by the alkali metal atoms can be seen in the literature, for instance, OM_3_ (M = Li, Na, and K) (Wang et al., 2011; Zein and Ortiz, 2011), NLi_4_ (Rehm et al., 1992), CLi_5_, SiLi_n_ (*n* = 1–5), CLi_6_ (Schleyer et al., 1983; Otten and Meloni, 2018), and BLi_6_ (Li et al., 2007). Next, a comprehensive work based on the *in silico* approach for binuclear superalkali cations having the formula M_2_Li_2k+1_ (M = F, O, N, C, and B for *k* = 1, 2, 3, 4, and 5, correspondingly) was reported by Tong et al. (2009). Furthermore, using the *ab initio*-based inspections, a series of binuclear superalkali cations M_2_Li_2k+1_^+^ (F_2_Li_3_^+^, O_2_Li_5_^+^, N_2_Li_7_^+^, and C_2_Li_9_^+^) was proposed by the same group (Tong et al., 2011). Then, focusing on the expansion from mono to binuclear superalkali cations, attempts have been made by Tong et al. (2012) in describing polynuclear superalkali cations YLi_3_^+^ (Y = CO_3_, SO_3_, SO_4_, O_4_, and O_5_) (Tong et al., 2013). The aforementioned remarkable cations/compounds offer a great deal of potential for use in the production of novel charge-transfer (CT) salts and cluster-assembled nanomaterials with customized characteristics, the reduction of carbon dioxide (CO_2_), and activation of very stable nitrogen molecules (N_2_), nitrogen oxides (NO), or as hydrogen (energy) storage materials as well as noble-gas trapping agents, ferroelectrics, catalysts, and nonlinear optical (NLO) response, among other things (Zhang et al., 2021b; Zhang et al., 2021a). These molecular species have the capability to reduce substances and can be employed to produce various cutting-edge building blocks of nanoscale materials having highly tunable features, particularly the CT salts. The superalkali cations can produce the CT salts such as a crystal salt Li_3_O^+^NO_2_^−^ consisting of a single singly charged superalkali cation (Li_3_O^+^) (introduced by Zintl and Morawietz, 1938) that alkali elements cannot because of the unfavorable energetics, the metal atom’s relatively high IP, or steric hindrance (Li et al., 2008; Tong et al., 2009). Subsequently, the real nature of sodium nitrate (NaNO_3_) was revealed to be sodium oxide nitrite, Na_3_O^+^(NO_2_)^−^ (Jansen, 1977). They can be utilized to create uncommon CT salts whose counterparts have a low electron affinity (EA). *In silico* design and characterization of superalkalis based on Zintl ions and the superatom compounds with an NLO response have been reported by some research groups along with Zintl clusters as a new class of superhalogens which can be seen in the literature (Reddy et al., 2018b, Reddy et al., 2018a; Sun et al., 2018; Sinha et al., 2022).

Design, synthesis, and potential applications of new materials consisting of large second-order NLO responses (involving optical data storage, optical computing, telecommunications, optical information processing, etc.) have gained increasing attention (Chemla and Zyss, 1987; Prasad and Williams, 1991; Bredas et al., 1994; Geskin et al., 2003). Several reports on organic molecules consisting of donor–acceptor- (D-A) or donor–π-conjugated bridge-acceptor (D-π-A) skeletons can be seen in the literature which demonstrate considerably large first hyperpolarizabilities and show high photovoltaic performance in developing new non-fullerene small-molecule acceptors (Marder, 2006; Wei et al., 2022). Such frameworks found with larger CT in the systems can establish substantial differences between the dipole moments of the ground state and the excited state as well as low-energy CT transitions (Xu et al., 2007). For example, a sequence of formal D-A chromophores is expected to show a significant NLO response and high stability when M_3_O (electron donor) interacts with its counterpart, BC_59_ (electron acceptor), and creates the CT dyads as M_3_O-BC_59_ (Tu et al., 2014).

Moreover, superalkali cations may interact with superhalogen anions, just like alkali metal cations, and this interaction is predicted to be stronger than the former due to the even lower IPs of the superalkalis. The ability of the superalkali clusters to replicate the chemical behavior of alkali metals makes them potentially useful as building blocks in the construction of innovative nanostructured materials, which is one of their most intriguing discoveries. In some situations, they can combine to form assemblies or super-atom compounds while retaining their identity, much like regular atoms, and thus can demonstrate unique chemical or tunable electronic characteristics. Examples include BF_4_-M (M = Li, FLi_2_, OLi_3_, and NLi_4_) (Yang et al., 2012), BLi_6_-X (X = F, LiF_2_, BeF_3_, and BF_4_) (Li et al., 2008), Na_2_XY (X = SCN, OCN, and CN; Y = MgCl_3_, Cl, and NO_2_) (Anusiewicz, 2010), and Al_13_(K_3_O) as well as Al_13_(Na_3_O) (Reber et al., 2007). As a result, it makes sense to study the characterization and prediction of the superalkali-based clusters. Recent investigations have attempted to suggest various unique superalkali species (Sun et al., 2016b, Sun et al., 2019; Tkachenko et al., 2019; Ye et al., 2022; Sun et al., 2022), including organic heterocyclic superalkalis [C_3_N_2_(CH_3_)_5_ acquired from a familiar aromatic heterocycle, pyrrole] (Reddy and Giri, 2016), organo-Zintl superalkalis (P7R4) (Giri et al., 2016), non-metallic superalkali cations [F_2_H_3_^+^, O_2_H_5_^+^, N_2_H_7_^+^, and C_2_H_9_^+^] (Hou et al., 2013), and aromatic superalkali species [MLi_n+1_^+^ aromatic superatom cations and Au_3_ core connected with pyridine (Py) and imidazole (IMD) ligands] (Sun et al., 2013; Parida et al., 2018) (33a, 33b).

As was earlier mentioned, the superalkali clusters are a common type of super-atoms that can function as alkali metal atoms, so they can construct an extended nanostructure when put together (Reber et al., 2007; Reber and Khanna, 2017). Additionally, it has been suggested that superalkali cations could act as hydrogen storage materials (Barbatti et al., 2002; Giri et al., 2011; Pan et al., 2012) and noble gas-trapping agents (Chakraborty et al., 2010; Pan et al., 2013a; Pan et al., 2013b). For instance, Giri et al. (2011) investigated the potential of the cationic superalkalis Li_3_^+^ and Na_3_^+^ for H_2_-binding. Additionally, it has been noted that the superalkali cation O_2_Li_5_^+^ can bind up to seven noble gas (Ng) atoms (Ng = He, Ne, Ar, and Kr) (Pan et al., 2013a) and up to 12 Xe atoms (Pan et al., 2013b).

The trinuclear Au_3_(IMD)_3_ [IMD = imidazole] combination turns aromatic, according to a recent work on the construction of a novel family of organometallic superalkali compounds (Gutsev and Boldyrev, 1982; Gutsev and Boldyrev, 1985; Parida et al., 2018; Tkachenko et al., 2019). Examining large systems such as fullerenes, the prediction of superalkali@C60 endofullerenes, their enhanced stability, and interesting properties have been reported comprehensively by Misra et al. in 2016 along with an understanding of the nanoconfinement effect on halogen bonding in (CH_3_Br···NH_3_)@C_60_ (Srivastava et al., 2016b; Srivastava et al., 2016a).

Even though superalkalis have advanced significantly over the past several decades, an overview of the recent advances in a variety of remarkable species appears to be missing in the literature (Sun and Wu, 2019; Pal et al., 2021). The most advanced developments in recent years, design/modeling, characterization in the framework of *in silico* approaches, and first-time use of superalkali-based novel materials are outlined in this review along with their potential applications. We attempted to share the predictions for their future growth in the hopes of pointing designers in the right direction as they continue to create and use such special species. We believe that this report can open new directions in the organized interpretation of the superalkali-based materials.

## Results and discussion

In this section, the authors have attempted to provide snippets on the structure, stability, and electronic features of various superalkali-based species such as NM_4_ (M = Li, Na, and K) superalkalis; *ab initio* exploration of the polynuclear K- and Na-based superalkali hydroxides as superbases; theoretical studies on the hydration effect on the superalkali cations, Li_3_^+^; and the role of the size and composition on the design of superalkalis. Moreover, in terms of the applications of a range of superalkali-based materials (i.e., high-performance NLO material/molecular switch), design and characterization (theoretically) of a novel series of D-A frameworks through superalkali–superhalogen assemblage, superalkalis doped with B_12_N_12_ nanocages, boron phosphide nanocages doped with superalkalis as novel electrides, and innovative inorganic aromatic mixed-valent superalkali electride, CaN_3_Ca, have been highlighted in this report. This report unravels a brief overview of the capturing of nitrogen by unveiling the potential of superalkali cation Li_3_^+^ and the reduction/activation of carbon dioxide with a superalkali. A short description of the modeling of aromatic organometallic superalkali complexes and superalkali ligands as the building blocks for aromatic trinuclear Cu(I)-NHC complexes, the nature of the aluminum trimer combined with different superatom clusters, superalkali–alkalide interactions, ion pairing in low-polarity solvents, and lead-based halide perovskites associated with superalkali species have been reported.

## Structural and electronic features of superalkali-based species

**FIGURE 1:**
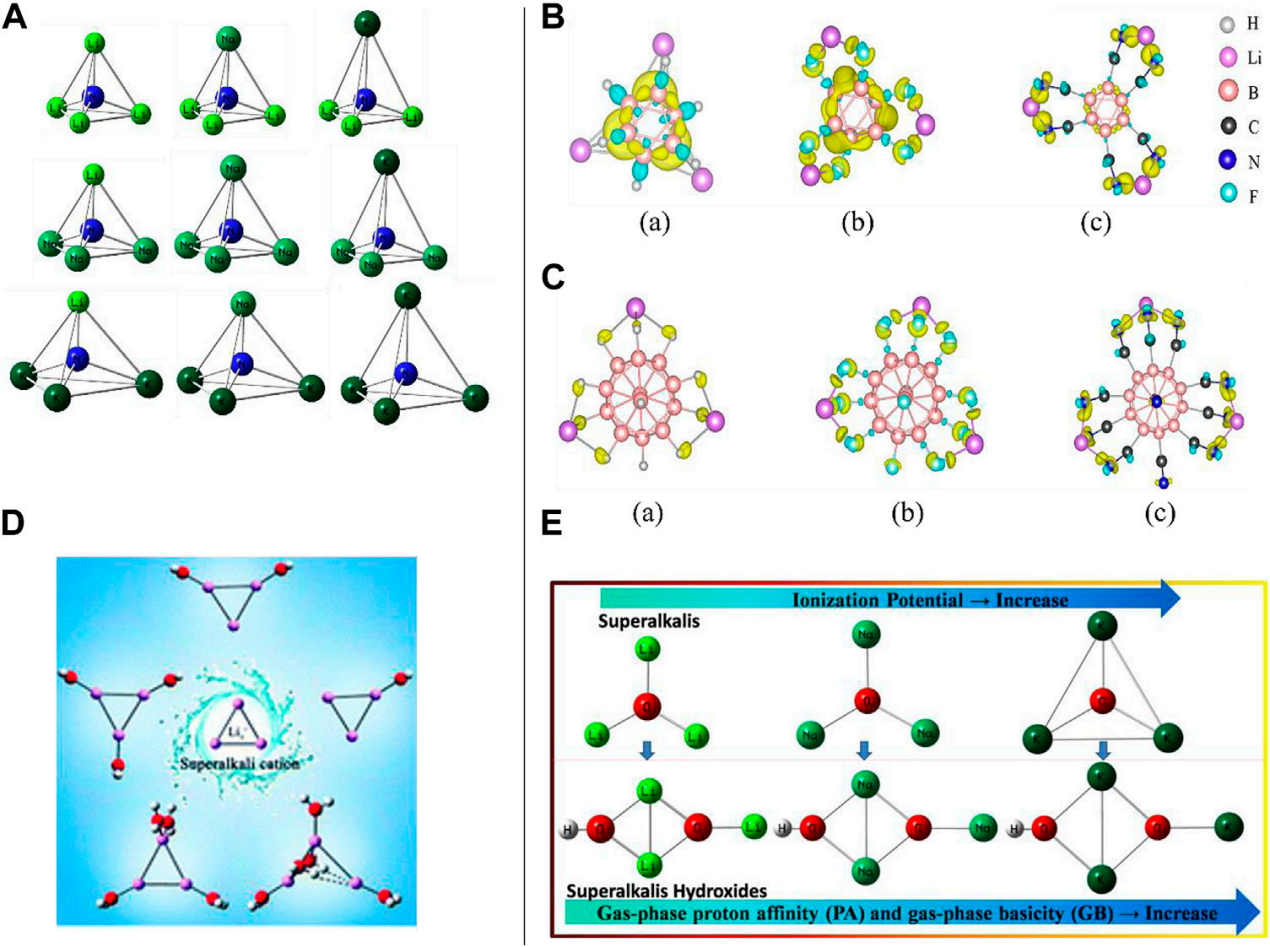
**(A)** Geometric structures of NM_4_ (M = Li, Na, and K) clusters reproduced from Zhang and Chen (2019); **(B)** charge density difference in **(A)** Li_3_B_6_H_6_, **(B)** Li_3_B_6_F_6_, and **(C)** Li_3_B_6_(CN)_6_ clusters reproduced from Banjade et al. (2021). **(C)** Charge density difference in **(A)** Li_3_B_12_H_12_, **(B)** Li_3_B_12_F_12_, and **(C)** Li_3_B_12_(CN)_12_ clusters (Banjade et al., 2021); **(D)** superalkali cation Li_3_^+^ with water molecules, Li_3_^+^(H_2_O)_n_ (*n* = 1–5), reproduced from Hou et al. (2018) with permission from the Royal Society of Chemistry; **(E)** a small series of hydroxides (XM_n+1_OH) where (XM_n+1_): superalkali; M (K and Na): alkali metal atom; n, maximal formal valence of the central atom X (F, O, and N), and *n* ≥ 1.

Superalkalis are known as super-atoms which can imitate the chemistry of atoms and can be used in the synthesis of novel materials with diverse interesting features. Using high theoretical-level Gaussian 3 (G3) calculations, Zhang and Chen proposed models of a small series of superalkali NM_4_ (where M = Li, Na, and K) (see Figure 1A) clusters consisting of homo- and hetero-alkalis, and studied their stability and electronic structures (Zhang and Chen, 2019). The calculated vertical IPs (3.22–3.74 eV) have been found to be smaller than that of the Cs atom having an IP value of 3.89 eV, and binding energies (BEs) and positive energies of the dissociation channel such as (NM_3_ + M’) confirmed the stabilities (thermodynamically) of all probed species where NLi_4_ was found to be the most stable among all the homo- and hetero-superalkalis. The order of the calculated BEs for the NM_4_ clusters was found to be NLi_4_ (−9.25 eV) > NNa_4_ (−5.88 eV) > NK_4_ (−1.92 eV), separately, where the size of the alkaline atoms has a substantial impact on the overall stability of the clusters when such atoms are combined, followed by ionic bond formation. Encouraged by the reports highlighted in this study, novel materials can be constructed by the previously designed and characterized (theoretically) species having high stability along with tetrahedral geometry. It is important to note that the structures of the NM_4_ species with homogeneous alkali atoms (like NLi_4_, NNa_4_, and NK_4_) have Jahn–Teller distortion-resistant T_d_ symmetry, while the clusters constructed from the heterogeneous alkali atoms have C_3v_ symmetry. Furthermore, the Frontier molecular orbital (FMO) approach appeared to show that the highest occupied molecular orbitals (HOMOs) of all such species are mainly spread over the complete clusters shown to diminish the repulsion among the electrons repelling each other. Their *ab initio* modeling approaches (MP2/MP4) described the highly symmetric structural parameters and the low IP values of the NM_4_ clusters from the electronic origin point of view.

It is to be noted that although significant signs of progress have been made in designing, synthesis, and characterization of superhalogens, there is still room for related studies on superalkalis. With the combination of superalkalis having low IPs and superhalogens having high EA, super-ions may be used as construction blocks of a novel series of supersalts having uses in multiferroic materials, metal-ion batteries, solar cells, and so on. Jena et al. thoroughly described the role of size and composition in the design of supersalts consisting of two dissimilar classes of clusters belonging to the *closo*-borane family, Li_m_B_n_X_n_ (*m* = 1–3; *n* = 6, 12; X = H, F, and CN) and Zintl ion family, Li_m_Be@Ge_9_, in the framework of the density functional theory (DFT) with hybrid exchange-correlation functional (ωB97xd) as well as Gaussian basis sets [6-311+G (d, p)] (Banjade et al., 2021). In this work, the stabilities of such a composition of clusters were regulated by the Wade–Mingos polyhedral skeleton electron pair theory (i.e., shell closure rule), and additionally, the jellium shell closure rule was deployed to understand the stability of the Be@Ge_9_ cluster. The analyzed IP values were well-connected to the EAs of the X ligands (the higher the EA, the larger the IP). They found that like the lowest IP value (2.84 eV) for Li_3_B_6_H_6_ among Li_3_B_n_X_n_ clusters, both the *closo*-borane family and Zintl ion-related clusters followed the same IP patterns; however, conversely and beyond the hope, the IP values were not reduced (*n* = 6–12) with enhancing the cluster volume followed by being weakly bounded. It should be noted that EAs of the B_n_X_n_ enhanced proceeding from *n* = 6 to *n* = 12, since the association of the Li atoms with the B_12_X_12_ cluster is stronger than that with the B_6_X_6_ cluster. Likewise, as the substitution of the X travels from the H atom to the F atom to the CN group in the Li_3_B_12_X_12_ clusters (X = H, F, and CN), the IP values are enhanced. The charge density difference (CDD) plots were visualized for all six species (three *closo*-borane and three Zyntl ion families). For example, in the case of Li_3_B_6_H_6_, the CDD showed that the electrons have been amassed in the area sandwiched between the three successive Li–B bonds and uniform diffusion of the electron near the Li–B bonding region (see Figure 1B), whereas a much larger charge accumulation between the Li and F atoms can be seen for the Li_3_B_6_F_6_ cluster. These findings were consistent with the structural features. Furthermore, electron accumulation along the Li–C bonds for the Li_3_B_12_X_12_ clusters demonstrated that the Li atoms were bonded to mostly the X atoms (see Figure 1C) which agreed with their geometries. Very importantly, however, there was an increment in the cluster volume and the F atom, as well as the CN group consisting of more electronegativity than the H atom, and stronger interactions between the cluster and the Li atoms have been observed. The IPs of the Zintl-ion family clusters were found to be higher than those in the *closo*-borane-family clusters since these behave like superalkalis. Very interestingly, it is noteworthy to mention that despite having the same electron count in both the Li_5_Ge_9_ and Li_3_Be@Ge_9_ clusters, the IP of the former is found to be smaller than that of the latter, implying that, indeed, composition matters therein.

Using the *ab initio* modeling approach, Li *et al.* reported an extensive study on the influence of hydration on the structure, stability (through energy decomposition analyses), and electronic features of the superalkali cation Li_3_^+^ in the framework of the MP2/6-311++(d, p) level of theory (Hou et al., 2018). Assuming all possible arrangements of H_2_O molecules around the Li_3_^+^ cation, a series of Li_3_^+^(H_2_O)_n_ (*n* = 1–5) structures (see Figure 1D) have been obtained where the Li_3_^+^ cation is observed to have a maximum of four coordination numbers. Interacting with five molecules of water, the CT of the Li_3_^+^ cation appeared to be critically irregular which was shown by natural population analysis (NPA), and as a result, the Li_3_^+^ cation lacks the ring conjugation and comes apart into the isomer of Li_3_^+^(H_2_O)_5_ having the lowest energy. Using the NPA tool, water ligands appeared to have a dominant role in the charge distribution of Li_3_^+^ along with the CT from the H_2_O ligand to the Li_3_^+^ skeleton. With the deployment of the polarization continuum model (PCM) tool in calculating the Gibbs free energies (∆*G*_r_^298^) at 298.15 K, the lowest-energy isomers of Li_3_^+^(H_2_O)_n_ (*n* = 1–5) are not appropriate for detaching spontaneously. It is important to mention that a coordination number of 12 has been vaticinated for the Li_3_^+^ cation associated with hydrogen clusters. The outcomes of the *in silico* approach revealed that structural and electronic integrity was maintained by the Li_3_^+^ cation followed by low-lying isomers of the Li_3_^+^(H_2_O)_1–4_ complexes, while in the lowest-energy structure of Li_3_^+^(H_2_O)_5_, it destroyed and left its superalkali individuality. Based on such findings, like Li^+^ in water clusters, the superalkali Li_3_^+^ cation contributes to the same maximum coordination number. Like the case of the lithium-ion hydrates, the localized molecular orbital energy decomposition approach showed that the electrostatic interactions play a prime role in the binding of the water molecules with the Li_3_^+^ cation. A sharp increase in the contribution of the exchange-repulsion energy can be seen when the number of water ligands arrives at five and even this exchange-repulsion energy surpasses that of the electrostatic term.

Very recently, computational designing and characterization of Na- and K-based superalkali hydroxides acting as superbases have been reported by S. K. Pandey for the first time (Pandey, 2021b). A new kind of hydroxide of the superalkalis (XM_n+1_OH) [where the fragment XM_n+1_ refers to the superalkali moieties, X (F, O, and N), and *n* ≥ 1] has been modeled, and inclusive computational experiments on such fascinating species have been performed using the framework of the *ab initio* method (see Figure 1E). To inspect the relative basic nature of such polynuclear superalkali hydroxides (SAHs) against the representative alkali metal hydroxide (KOH, NaOH, and LiOH) along with the Li-related SAHs, the *ab initio* findings demonstrated that both the Na- and K-based SAHs are stronger bases than the LiOH and Li-related SAHs which is due to the larger gas-phase proton affinity (**PA**) and gas-phase basicity (**GB**) values of the Na- and K-related SAHs. Noticeably, the highest **PA** (1168.4 kJ/mol) and **GB** (1146.9 kJ/mol) of the OK_3_OH base revealed its strongest basicity nature among all the existing strong bases and superbases along with the proposed K- and Na-based SAHs. The values adequately surpassed the **ΔPA** (142.1 kJ/mol) and **ΔGB** (146.9 kJ/mol) values of the threshold values (PA: 1026.3 kJ/mol and GB: 1000 kJ/mol) of a very popular IUPAC-defined superbase (DMAN).

To probe the structure, stability (bonding features), and electronic properties of the SAHs, the popular noncovalent interaction (NCI)-plot and quantum theory of atoms in molecules (QTAIM) tools were used for a variety of chemical to biochemical species to materials along with exploration of the computational studies on the HAP biomaterials including the first-principle DFT, *ab initio* modeling, and molecular dynamics simulations (Awasthi et al., 2021a; Awasthi et al., 2021b; Pandey, 2021a; Pandey and Arunan, 2021). In this report, new insights into the basicity features were facilitated by an *ab initio* modeling approach. Design, fabrication, and synthesis of the theoretically explored SAHs may lead to providing an alternative pathway for the experimentally availing applications.

## High-performance NLO response material/molecular switch

**FIGURE 2:**
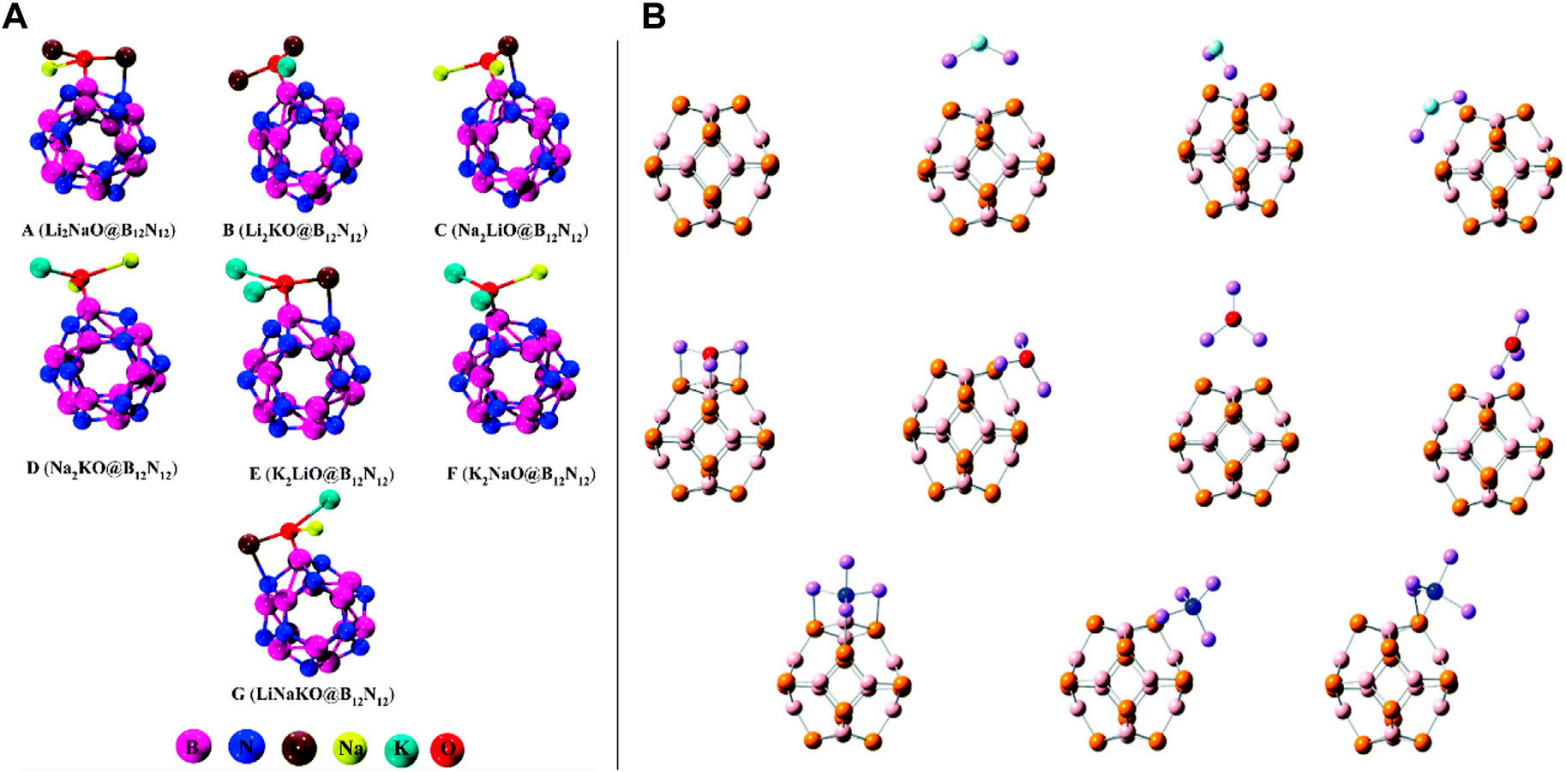
**(A)** Optimized geometries of the most stable complexes of mixed-superalkali-doped B_12_N_12_ adapted from Bano et al. (2022) with permission from the Royal Society of Chemistry; **(B)** optimized structures of C_6_Li_6_-nCO_2_ complexes (front and side views) for *n* = 1–6 adopted from Srivastava (2019b) with the permission of Wiley.

Very recently, Bano et al. presented theoretical work where they showed that the mixed superalkalis (Li_2_NaO, Li_2_KO, Na_2_LiO, Na_2_KO, K_2_LiO, K_2_NaO, and LiNaKO) could be a better alternative than the pure superalkalis for B_12_N_12_ nanocages in designing hi-tech NLO materials (Bano et al., 2022). The optimized structures of the mixed-superalkali-doped B_12_N_12_ complexes can be seen in Figure 2A. Throughout the computational experiments, the structure, stability, electronic, and NLO response features of superalkali cluster-mixed B_12_N_12_ nanocages were examined. They have chosen a total of seven (**A–G**) thermodynamically stable designed complexes consisting of very high interaction energies ranging from −98.02 kcal/mol to −123.13 kcal/mol, which were compared with the previously reported Li_3_O@B_12_N_12_ having an interaction energy (E_int_) as −92.71 kcal/mol along with the narrow variation of the HOMO-LUMO energy gaps (from 3.36 eV to 4.27 eV) compared against the pristine B_12_N_12_ (11.13 eV) species. This group confirmed the CT phenomenon occurring in such probed complexes which were acquired from the NPA technique, non-bonding interactions between the doped superalkalis and nanocages followed by the QTAIM, as well as NCI-plot tools. The maximum absorbance was found in the near-infrared (IR) region ranging from 1,076–1,486 nm which appeared to be transparent in the ultraviolet (UV) region. The computed first hyperpolarizability (*β*_tot_) of complex C was analyzed as 1.7 × 10^4^ au which was much greater than that of the pure Li_3_O superalkali-doped B_12_N_12_ complex (3.7 × 10^4^ au) perceived from the same theoretical level of approach (by Sun *et al.* in 2016). As in nanoelectronics, designing and synthesizing stable and high-performance NLO materials is the foremost precedence for the scientists and researchers; an increased NLO response was detected in this work by a large second hyperpolarizability. To gain more insights into such complexes, they also computed hyper-Rayleigh scattering (6.71 × 10^10^ au), second harmonic generation (1.17 × 10^10^ au), and electro-optical Pockels (3.29 × 10^10^ au) effect along with the maximum obtained values of the electric field-induced second harmonic generation (3.46 × 10^10^ au) and electro-optic-dc-Kerr effect (3.96 × 10^11^ au) at 1,064 nm wavelength. At the same wavelength for all A-G complexes, significant increments in quadratic nonlinear refractive index (*n*_2_) values were seen along with the largest value of 3.35 × 10^–8^ cm^2^·W^−1^. The theoretical findings based on the ωB97XD/6-31G (d, p) level of approach indicated that such mixed superalkali-doped nanoclusters could have broad applications in designing, fabrication, and characterization of hi-tech optoelectronics (i.e., high-performance NLO materials).

However, various procedures can be seen in the literature in designing high-performance NLO materials; the NLO response of three series (a total of 10 isomers, **I**–**X**) of theoretically designed compounds Li_2_F@B_12_P_12_, Li_3_O@B_12_P_12_, and Li_4_N@B_12_P_12_ having three (**I**–**III**), four (**IV**–**VII**), and three (**VIII**–**X**) isomers, correspondingly, was inspected thoroughly using the DFT approach (see Figure 2B) (Ullah et al., 2019). This research work explored the effects of superalkali (Li_2_F, Li_3_O, and Li_4_N) when these were doped on the B_12_P_12_ nanocage. The computational outcomes showed that most of the complexes [all isomers of the Li_2_F@B_12_P_12_ and Li_3_O@B_12_P_12_ model compounds (**I**–**III** and **IV**–**VII**) along with one isomer of the Li_4_N@B_12_P_12_ complex (**IX**)] possessed excess electrons, whereas only two isomers of the Li_4_N@B_12_P_12_ (**VIII** and **X**) compound were found to be inorganic electrides. It was computationally inspected that the superalkalis were chemisorbed on the boron phosphide nanocage and all such formed nanocage complexes were found to be quite stable which was confirmed by their calculated BEs. There was substantial enrichment in the first hyperpolarizability values of the system when doping a B_12_P_12_ nanocage with the superalkali. Isomer **III** of the Li_2_F@B_12_P_12_ system showed the largest first hyperpolarizability (3.48 ×10^5^ au) and lowest the HOMO-LUMO gap (1.46 eV) among the three isomers (**I**–**III**) along with good transparency in the UV region, showing that the superalkali Li_2_F provided better hyperpolarizability increment than the Li_3_O and Li_4_N superalkalis when associated with the B_12_P_12_ nanocage. In addition, the influences of several superalkalis and various doping locations on the NLO response were comprehensively examined for all three variants of model systems in the framework of the quantum chemical calculations using the DFT approach. This work will promote prospective uses of the C_60_-like B_12_P_12_-based nanostructures doped with the superalkali and novel electronic nanodevices, and hi-tech NLO materials consisting of good UV transparency could be designed by the employment of new excess electron systems and electrides (**VIII** and **X**).

**FIGURE 3:**
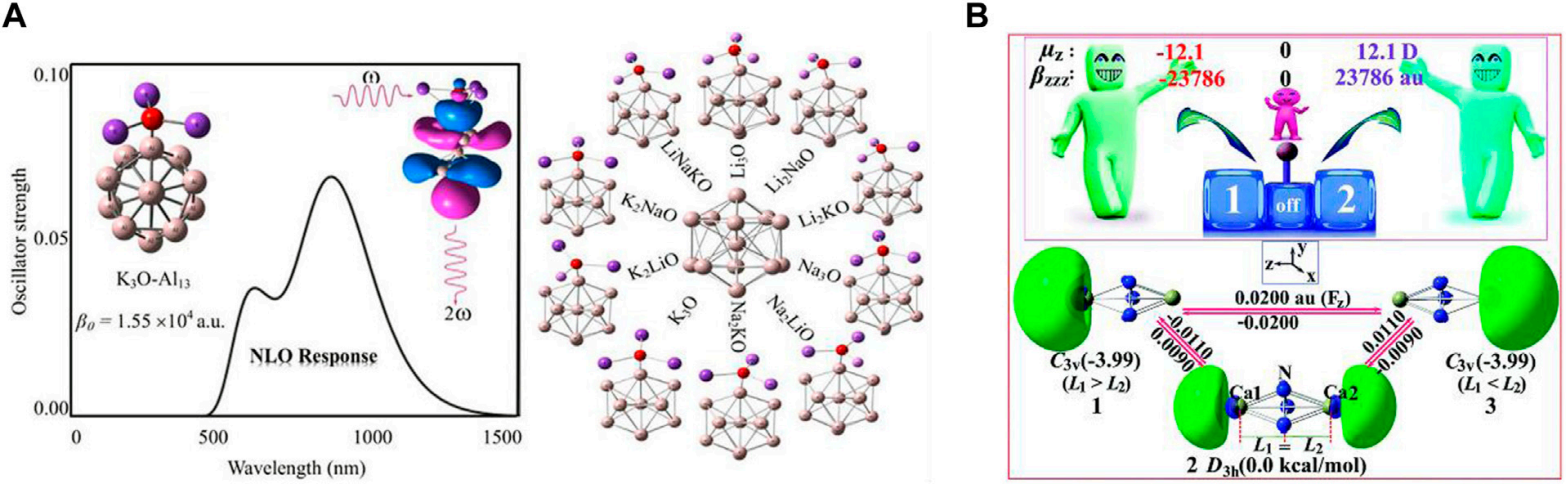
**(A)** Structures of superalkalis, M_3_O (where M = Li, Na, and K) associated with the Al_13_ nanocluster reproduced with permission from Omidvar (2018); **(B)** different configurations (1, 2, and 3) of the CaN_3_Ca molecule adopted from Wang et al. (2020) with permission from the Royal Society of Chemistry.

As several research groups have focused on designing novel superatoms with high NLO responses, A. Omidvar reported a range of representative D-A model skeleton species having elevated NLO responses through bonding the superalkali-like M_3_O (K_3_O, Na_3_O, Li_3_O, Li_2_KO, Li_2_NaO, Na_2_KO, Na_2_LiO, K_2_LiO, K_2_NaO, and LiNaKO) blended with the superhalogens (Al_13_ cluster) (see Figure 3A) (Omidvar, 2018). These superalkalis consist of a low IP to the superhalogen Al_13_ having a larger EA. Additionally, the electric field gradient tensors of the ^17^O nuclei and the natural bond orbital (NBO) analysis-based charges of the M_3_O superalkalis were also computed. In this study, he represented the theoretical signatures for the feasibility of employing the super-atoms Al_13_ and M_3_O as construction blocks to construct nanomaterials with strong NLO responses. He observed that the IP and ^17^O nuclear quadrupole resonance parameters of the M_3_O superalkalis have been efficiently affected by the M ligands. The electron transfer phenomenon in such types of super-atoms in the bonding superalkalis is responsible for the competent narrow HOMO-LUMO gap as well as increasing the first hyperpolarizability remarkably in the pristine Al_13_ cluster. Significant CT takes place from the M_3_O component to the Al_13_ assembly. It was observed that the data based on the η_Q_ parameters of the ^17^O nuclei significantly increase when the pure ligands were substituted with the doped ligands in the M_3_O superalkali species. The Q value (an asymmetry parameter) provides evidence of the chemical bonds taking part therein. Importantly, according to the findings of this investigation, the electropositivity of the alkali ligands was shown to play an important role in the determination of the IPs of the superalkalis. Moreover, a comprehensive exploration of the NLO response of the super-atoms M_3_O-Al_13_ can be seen that is altered by the oriented external electric fields. When the imposed oriented external electric field is enhanced along the CT direction (M_3_O → Al_13_) ranging from zero to a critical external electric field, a gradual increment in the first hyperpolarizability can be seen for the superatom compounds which appears to be used as an effective approach based on the theoretical findings.

Using quantum mechanical approaches, a total of three configurations [two C_3v_ point groups (**1** and **3**) and one D_3h_ point group (**2**)] of trigonal bipyramidal (TBP) CaN_3_Ca structures were chosen as models, and the role of the NLO response was analyzed by aiming at pioneering hi-tech single-pole double-throw (SPDT) NLO molecular switches (Wang et al., 2020). One marginally longer and one shorter vertical Ca-N_3_^3−^ length was found in the case of both C_3v_ configurations (**1** and **3**), whereas there were two identical bond lengths of Ca-N_3_^3−^ in configuration **2**. The *ab initio* (MP2/6-311+G (3df) method-based equilibrium structures of the chosen CaN_3_Ca species can be seen in Figure 3B where the Ca atoms are located over and under the usual triangular N_3_ ring. It should be noted that energy configurations **1** and **3** are 3.99 kcal/mol smaller than that of configuration **2** at the CCSD(T)/6-311+G (3df) level of theory which illustrates that the former two configurations (**1** and **3**) have a larger thermal stability than configuration **2**. Moreover, in the case of the configuration **2**, the excess electrons equally occupied the two hemispherical orbital lobes (HOMOs) positioned over the two extremes of the CaN_3_Ca molecule, while in the case of the other two configurations (**1** and **3**), the excess electrons mainly occupied one hemispherical orbital lobe (HOMO) (located on the left side for **1** and right side for **2**) of the CaN_3_Ca species. Importantly, Figure 3B also confirms that a uniform external electric field (EEF) triggered a reversible configuration conversion between **1** (L1 > L2) and **3** (L1 < L2). Hence, by deploying an appropriate external homogeneous electric field, interconversion between **1**, **2**, and **3** can be realized easily. All these species belonged to the novel alkaline-earth-based aromatic mixed-valent superalkalis along with acting as fascinating electrides. Very importantly, the electronic structure of the salt-like species containing configuration **2** showed a delocalized structure in the form of e^0.5−^···Ca^2+^N_3_^3−^Ca^2+^···e^0.5−^ demonstrating class III-type mixed-valent superalkali electrides, whereas the other two configurations **1** and **3** were confirmed as the rare inorganic Robin–Day class II-type structure consisting of localized redox centers. Moreover, comparison outcomes of remarkable static and dynamic first hyperpolarizabilities of all three configurations have been reported since all three were excellent candidates for SPDT NLO molecular switches.

Finally, using the *ab initio* modeling approach, Zhang *et al.* showed that the phenalenyl radical and M_3_ ring (M_3_–PHY, M = Li, Na, and K) stacked with parallel and vertical geometries are potential applicants for molecular switches which can exist in both superalkali electrides and superalkalides (Yi et al., 2022). A range of similar kinds of interesting research studies can be seen in the aforementioned literature. In this work, they have shown that M^δ−^-M_2_^(1−δ)+^-PHY^-^ acts as a superalkalide, whereas the e^−^⋅⋅⋅M_3_^+^-PHY acts as a superalkali electride where the former may isomerize to the latter using suitable long-wavelength irradiation and the latter may isomerize to the former with appropriate short-wavelength irradiation. In this report, the researchers have combined both electride and alkalide characteristics in one molecular switch and concluded that both forms demonstrated an excellent functioning NLO response.

## Capturing carbon dioxide/nitrogen/hydrogen

**FIGURE 4:**
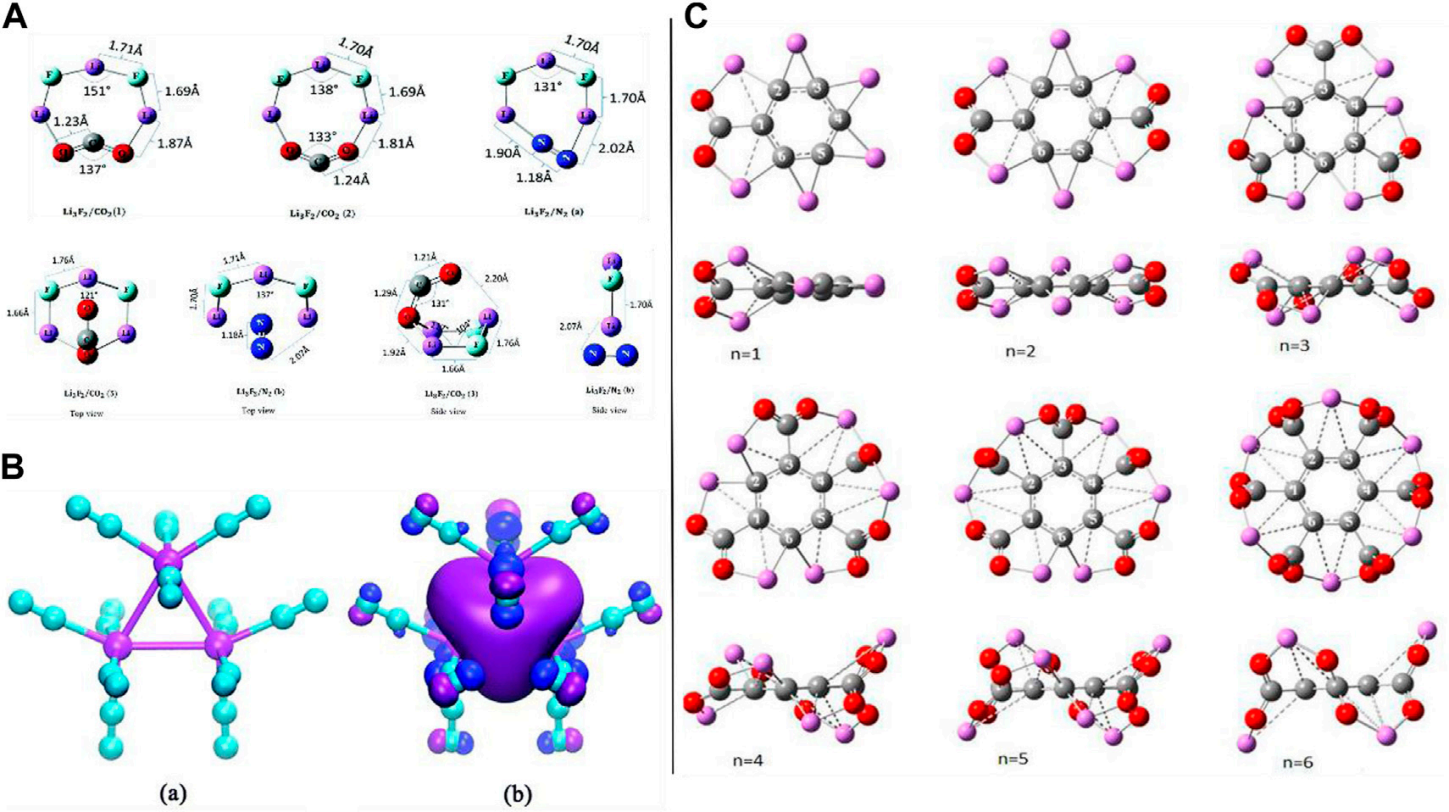
**(A)** Optimized geometries of neutral planar clusters (top) and neutral non-planar clusters (bottom) adapted from Park and Meloni (2017) with permission from the Royal Society of Chemistry; **(B) (a)** optimized structure of the Li_3_^+^(N_2_)_12_ complex and **(b)** its HOMO 3D isosurface map adapted from Yu et al. (2020) with permission from the Royal Society of Chemistry; **(C)** optimized structures of C_6_Li_6_–nCO_2_ complexes (front and side views) for *n* = 1–6 adopted from Srivastava (2019b) with the permission of Wiley.

Reduction (*via* electron transfer i.e. CT phenomenon) of carbon dioxide (CO_2_) by the capability of the superalkali Li_3_F_2_ has been shown by Park and Meloni (2017). The equilibrium structure of neutral planar and non-planar clusters trapping the CO_2_ and N_2_ molecules can be seen in Figure 4A. To attain trustworthy outcomes on the structural and energetics/stability of the species, a composite CBS-QB3 model was deployed. The association of CO_2_ with the Li_3_F_2_ superalkali was examined by scanning the potential energy surface (PES) which led to the formation of their transition states (TSs) and minima. Structural alterations were exposed in the terms of spin density and charge flow. The large BE (ionic interactions between the Li_3_F_2_ and CO_2_ having the largest BE (163 kJ/mol) among the three optimized structural isomers), CT phenomenon, and the HOMO-LUMO gap explained the energetics and stability of the Li_3_F_2_/CO_2_-associated species. It is worth mentioning that the largest determined BE (163 kJ/mol) was found to be significantly larger than that of the superhalogen cluster Sb_3_F_16_/CO_2_ reported by Czapla and Skurski (2017). The choice of the Li_3_F_2_ toward the CO_2_ has been taken using the same level of approach by which computational experiments on the association of the most copious atmospheric N_2_ gaseous molecule have been conducted (a very small chemical affinity of the Li_3_F_2_ for N_2_). In the case of the N_2_ association with the superalkali Li_3_F_2_, the calculated BE value was only 51 kJ/mol, which reveals that the capability of the superalkali Li_3_F_2_ for CO_2_ reduction has high selectivity over the N_2_ molecule. CO_2_ is actively reduced by the superalkali Li_3_F_2_ followed by transferring an electron as well as geometric change which brings up the CO_2_ molecule in a bent shape, and it can be probably used in transforming CO_2_ into fuel.

With the deployment of the MP2/6-311+G(d) level of approach, an investigation was conducted by Yu et al. (2020) to obtain new insights into the capability of the superalkali cation Li_3_^+^ for capturing N_2_ gas as well as its behavior in gaseous nitrogen. To reduce inter-ligand Coulombic repulsion, the N_2_ molecules (i.e., a maximum coordination number of 12 was envisaged for the trinuclear Li_3_^+^ because every vertex Li can capture four N_2_ molecules) approached to attach at various apexes of the trinuclear Li_3_^+^ core of the Li_3_^+^(N_2_)_n_ (*n* = 1–7) complexes which were confirmed by the structure, stability, and electronic feature analyses. It is worth to mention that only in the case of the lowest-lying series of the Li_3_^+^(N_2_)_n_ complexes (where the number “n” ranges from 1 to 4) complexes, the trinuclear Li_3_^+^ core retains its superatom dignity using the NPA and FMO approaches. Importantly, the interaction between the Li_3_^+^ with N_2_ [for example, it reached up to −24.5 kcal/mol for the Li_3_^+^(N_2_)_4_ complex] is stronger than that with H_2_ but weaker than that with H_2_O molecules. The difference in the Gibbs free energies for the same series of model complexes (through possible fragmentation channels) specified the thermodynamic stability of the Li_3_^+^ in the (N_2_)_n_ clusters. As electrostatic interaction plays a dominant role over the polarization component in the case of the Li_3_^+^(H_2_O)_n_ complexes, a different case was found in the construction of the Li_3_^+^(N_2_)_n_ complex having a non-covalent nature where the electrostatic and polarization components contributed almost equally. Furthermore, they concluded that the superalkali trinuclear Li_3_^+^ cation is a much better candidate than its other heavy alkali metal cations (Na_3_^+^ and K_3_^+^) in terms of capturing N_2_ molecules which is due to a larger BE and favorable structural features for the Li_3_^+^(N_2_)_n_ complexes as compared to the N_2_ associated with the Na^+^ and K^+^ cations. Moreover, by resemblance, the Li_3_^+^ superalkali cation (along with retaining its identity) has been predicted to have 12 maximum coordination numbers in the nitrogen clusters whose structure (see Figure 4Ba) was assured by the *ab initio* molecular modeling approach with the deployment of the MP2/6-311+G(d) level of theory and the local minima was confirmed by the frequency calculations. Analogous to the isolated Li_3_^+^ alkali metal cation, a delocalized s-type bonding HOMO was detected in the case of Li_3_^+^(N_2_)_12_ which can be seen in Figure 4Bb. The computed NMR parameters (chemical shifts ranging from −95.5 to −94.1 ppm and −66.8 to −64.0 ppm for the ^15^N1 and ^15^N1′ nuclei, correspondingly) can indeed provide significant information for the experimental classification of the complexes. Of course, this work provided an in-depth understanding of the mechanism of binding interactions in the Li_3_^+^(N_2_)_n_ complexes and explored the superalkali-based nitrogen-gas-trapping agents by spurring more computational and experimental attempts.

As CO_2_, with the property of trapping greenhouse gases, has a crucial role in the global carbon balance purpose along with its conversion into fuel, a very recent *in silico*-based report by H. Srivastava and A. K. Srivastava on the activation of CO_2_ by various types of superalkalis can be seen in the literature where they showed comprehensive and interesting theoretical outcomes (see Figure 4C) (Srivastava and Srivastava, 2022). Some studies on the superbases which were designed with the help of superalkalis can be seen in the literature (Srivastava and Misra, 2015, Srivastava and Misra, 2016). Because of the superalkalis, for example, typical superalkalis (FLi_2_, OLi_3_, and NLi_4_), special superalkalis [Al_3_, Mn(B_3_N_3_H_6_)_2_, B_9_C_3_H_12_, and C_5_NH_6_], binuclear superalkalis (Li_3_F_2_), non-metallic superalkalis (O_2_H_5_ and N_2_H_7_), and polynuclear superalkalis (Al_12_P, N_4_Mg_6_M) having lower IP than those of the alkali atoms and can easily transfer an electron (due to its hypervalent nature) to the CO_2_ molecule, these species possess strong reducing power and play an imperative role in altering the geometry of CO_2_ (bent shape) from its linear structure by donating an electron to it, which have been reported based on the quantum chemical methods. Examining different theory-based reports, it was observed that the extent of the CT (electron transfer) is mainly dependent on the size, geometrical, and electronic features as well as the (IPs) of the superalkali species. *In silico*-based investigations and discussions on the activation of CO_2_ by a small Li_3_F_2_ cluster inside the Buckminsterfullerene can also be seen in the literature. It was also found that the CO_2_ molecule is not only activated by the C_6_Li_6_ molecule but also a total of six CO_2_ molecules can be trapped by the same species. From this report, Srivastava et al. came up with concluding remarks that the reported theoretical findings imply that superalkalis could be utilized as an economical catalyst for the activation of a CO_2_ molecule, and since the fuel can be formed by the activated CO_2_ ion like methanol (CH_3_OH) followed by the hydrogenation reaction. Moreover, Srivastava et al. (2022) have reported various computational experiments using the DFT as well as *ab initio* modeling approaches on the superalkali cations (X = F, O, and N) with methyl ligands, superalkali behavior of ammonium and hydronium cations, and many more studies (Srivastava, 2019b). Moreover, introducing the superalkalis, an *ab initio* study on single- and double-electron reductions of CO_2_ was reported in 2018 (Srivastava, 2018).

## Complex formations and interactions involved therein

**FIGURE 5:**
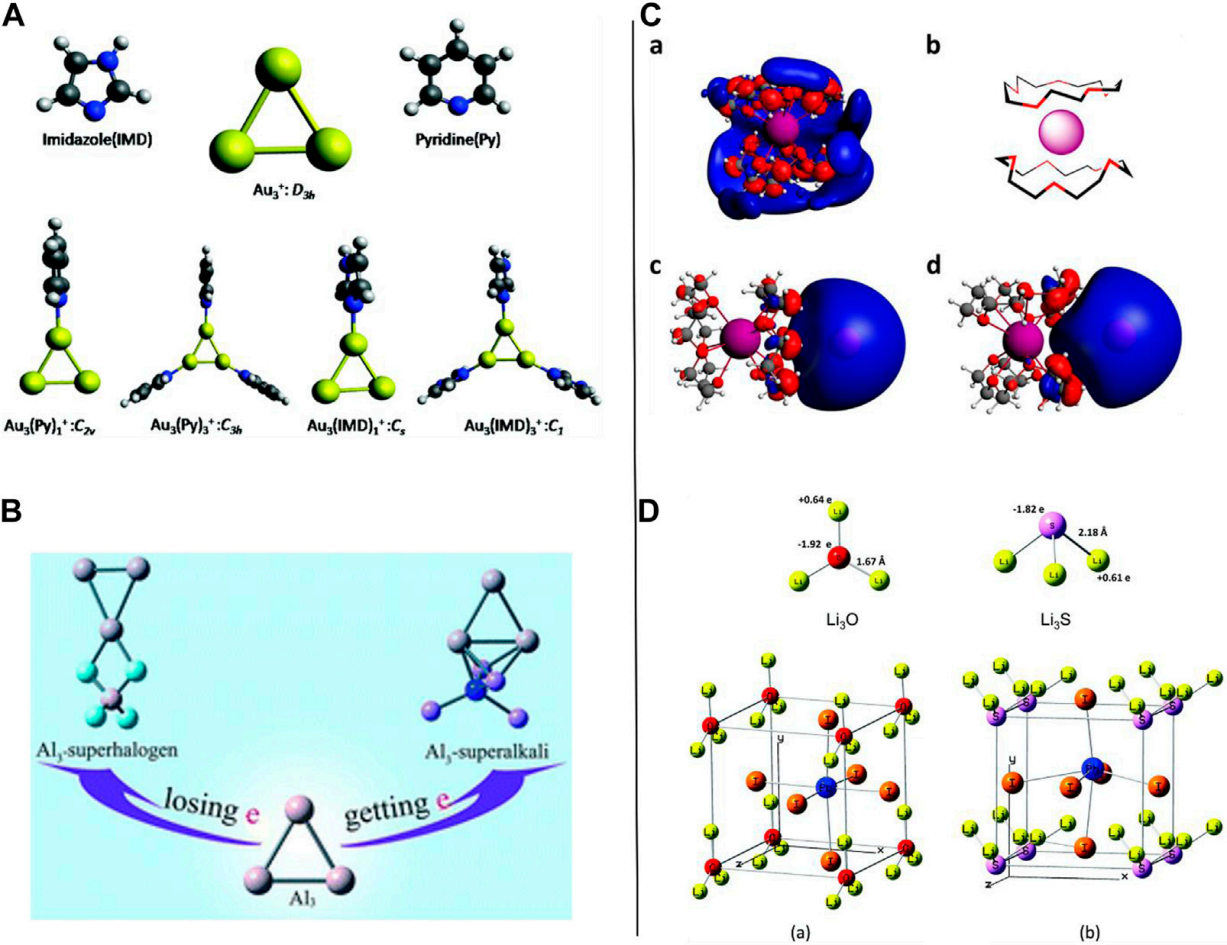
**(A)** Optimized geometries of the ligands and the Au_3_-coupled complexes reproduced with permission from Parida et al. (2018); **(B)** representative Al_3_-M and Al_3_-X superatom compounds where M is a (super)alkali (M = Li, FLi_2_, OLi_3_, and NLi_4_) and X is a superhalogen (X = F, LiF_2_, BeF_3_, and BF_4_) reproduced from Yang et al. (2018) with permission from the Royal Society of Chemistry; **(C)** computed SOMO/HOMO 3D isosurfaces of the superalkali K-15-crown-5_2_ model **(a)** and superalkali−alkalide (K-15-crown-5_2_)^δ+^(Na)^δ−^ with the alkalide in the axial reproduced from Riedel et al. (2021) **(c)** and equatorial **(d)** positions with respect to the sandwich complex [as illustrated in the form of a cartoon **(b)**]; **(D)** optimized geometries for **(a)** Li_3_O and **(b)** Li_3_S (top); cubic ABX_3_ perovskite structures with superalkali clusters occupying the A sites, Pb atoms occupying the B sites, and I atoms occupying the X sites (bottom) adapted from Paduani and Rappe (2017) with permission from the Royal Society of Chemistry.

To begin with the idea of superalkalis, complexes are superalkalis with a lower IP than the corresponding alkali and alkaline earth metals; Parida et al. reported work on the computational designing of aromatic organometallic superalkali complexes using the first principle calculations (see Figure 5A) (Parida et al., 2018). A trigonal all-metal core coupled with imidazole (IMD) and pyridine (Py) has been proposed which facilitate the probable existence of a superalkali complex. Low IEs (as compared to the Cesium atom) were found for the organometallic complexes, Au_3_(IMD)_3_ and Au_3_(Py)_3_, which imitated the general features of a superalkali very well. All the superalkali clusters revealed sp^2^ hybridization, and therefore, a planar geometry all along with the Au_3_^+^ ring showed a “doubly aromatic” character. The HOMO-LUMO gap value of the Au_3_(Py)^+^ species (4.11 eV) was found to be greater than that of the Au_3_(IMD)^+^ (3.84 eV). As some of the organometallic complexes were capable of showing good NLO features using the static first-order hyper-polarizability calculations, it also appeared that the characteristics of such Au-based complexes are like the features of a superalkali. Such outcomes concluded that various coinage metal-based superalkali complexes having broad applications can be synthesized in the laboratory.

Combinations of aluminum trimer and two distinct forms of the representative superatom clusters [(super) alkalis: M = F, FLi_2_, OLi_3_, and NLi_4_; and (super) halogens: X = LiF_2_, BeF_3_, and BF_4_)] were chosen, and the diverse structural, stability (interactions involved in the Al_3_-M and Al_3_-X superatom compounds), and electronic features were theoretically explored by Yang et al. (2018) at the MP2/6-311+(3df) level of theory. The Al_3_-M and Al_3_-X compounds (see Figure 5B) showed different structures where point-to-side interactions were seen between the Al_3_ and X components of the Al_3_-X compound, providing the most favorable bonding pattern while the least preferable was face-to-face. In the case of Al_3_-M systems, Al_3_ favored interacting with the FLi_2_ and Li (super) alkalis *via* its ring plane, whereas it favored binding with NLi_4_ and OLi_3_ superalkalis over the Al–Al edge. Interestingly, the Al_3_ moiety of the Al_3_-X species acts as the cation (electron donor), whereas it acts as an anion (electron acceptor) in the Al_3_-M compound assured by the NPA approach. Enhanced CT was detected between the Al_3_ component and the super-atoms (M and X) in both polar and nonpolar solvents. The HOMO-LUMO gap values, BEs, and bond energies were calculated to be large which confirmed the stability of such blended species (Al_3_-M and Al_3_-X compounds) supported by strong interactions taking part between the Al_3_ and super-atoms (M/X). Importantly, the solvent effect has an important role in the case of the Al_3_-M (especially, the Al_3_-NLi_4_) compounds where it was found that such species were better stabilized in the presence of the solvent molecules, whereas the outcomes were not significant for the stability of the Al_3_-X compounds. Moreover, the Al_3_^+^ ring exhibited different aromatic characters (*σ* and *π* aromaticity) when associated with diverse superhalogen anions either in the gas or in liquid phases.

As it has been conventionally considered that the anionic alkali metals in the solution possess a gas-like and unperturbed nature recently, Barrett *et al.* reported experimental and theoretical findings on superalkali–akalide interaction-related studies which assisted ion pairing in alkalide solutions in solvents having low polarity (Riedel et al., 2021). The experimental evidence (based on coherent neutron scattering, dielectric spectroscopy, and concentration-dependent macroscopic ionic conductivity) of the observation and influence of ion pairing of alkalides in the solution agreed with each other. Furthermore, to understand the structural, stability, and electronic features, the DFT studies for a range of possible superalkali–alkalide complexes chosen as models were carried out in the framework of *ab initio* simulations, including the superalkalis and superalkali dimers where such species were revealed to play the dominant role in the solution. The visualized singly occupied molecular orbital (SOMO)/HOMO 3D isosurface maps of the superalkali K-15-crown-5_2_ model (a), superalkali−alkalide (K-15-crown-5_2_)^δ+^(Na)^δ−^ associated with the alkalide in the axial (c), and equatorial (d) sites concerning the sandwich complex are exemplified in the form of a cartoon (b) as shown in Figure 5C. The conformational flexibility of the alkalide systems measured by the temperature-dependent alkali metal NMR spectra appeared to show both reversible perturbation and thermally triggered demonstrating a complex replacement mechanism for the ion-paired moieties. The outcomes of their research work facilitated a picture of alkalide moieties acting as a gas-like ion in the solution and attached a great significance to the interactions of the alkalide with its complex counteraction (superalkali) which could be chemically regulated and established by contemplating the interactions between the superalkali and alkalides.

Recently (in 2021), to synthesize aromatic trinuclear Cu(I)-NHC complexes constructed from superalkali ligands as a building block, a variety of NHC (N-heterocyclic carbene) ligands based on imidazole and benz-imidazole have been computationally designed by Giri *et al*. Six different ligands were chosen based on the pyridazine, pyrimidine, and pyrazine acting as the skeletons (Parida et al., 2019). According to the outcomes of the calculated vertical electron affinity, all of the ligands chosen in this work seem to be of the superalkali kind of molecule which has excellent NLO properties. The Cu(I)@NHC complexes were formed by the NHC ligands consisting of trinuclear Cu_3_ as the central moiety. From the contribution of canonical molecular orbitals toward the computed nucleus independent chemical shift (NICS) values, the trinuclear Cu_3_ ring appeared to show high σ- and low π-aromaticity. Depending on the various ligand environments, the aromatic features were altered along with other reactivity parameters such as electrophilicity and hardness. According to their theoretical findings, a more reactive and less aromatic complex can be produced by the pyrazine-based NHC ligand as opposed to pyridazine and pyrimidine-based NHCs. Like the BH_3_ molecule, the inspected super-atomic clusters exhibit sp^2^ hybridization based on the natural localized molecular orbitals (LMOs). Very interestingly, the UV-Vis spectrum calculations using the first principle study in the acetonitrile solvent showed a blue shift. It is believed that such modeled and theoretically explored complexes can be simply synthesized and utilized as catalysts in advanced synthetic chemistry.

The influence of variations in the ionicity of CT and bonding mechanism on the geometrical and electronic features of novel lead-based halide perovskites formed by introducing the superalkali species was reported by Paduani and Rappe in 2017 with the employment of the scalar relativistic DFT approach (Paduani and Rappe, 2017). In this work, the authors used two superalkalis such as Li_3_O [see Figure 5Da] and Li_3_S [see Figure 5Db] which were replaced by the Cs atoms in the CsPbI_3_ having a cubic structure which were detected to be slightly tetragonally distorted, and the band gap values of both superalkali-based species were much lower than that of the cubic CsPbI_3_. The *in silico*-based outcomes facilitated encouraging performance which can have applications in optoelectronics, for instance, as long-wave infrared (IR) sensors and thermoelectrics. They found that the incorporation of suitable superalkali species at the cationic A-positions in the ABX_3_-type (CsPbI_3_) structure can have the capability to tune the band gap of such species. Smaller band gaps (mainly occurring from the effective hybridization between the Pb and Li s-states at the top of the valence band) for the minimum-energy (i.e., equilibrium) crystal structures of both the [Li_3_S]PbI_3_ and [Li_3_O]PbI_3_ compounds were analyzed as 0.41 eV (indirect) and 0.36 eV (direct), correspondingly, where the latter is slightly larger than the former. The [Li_3_O]PbI_3_ compound was hoped to be more resistant to water exposure which was confirmed by the cluster calculations. In comparison to the CsPbI3 species, harsh alterations can be seen in the shape of both valance and conduction bands when the chemical environment was changed in the case of the Pb-halide perovskite structure. Because of the construction of the delocalized energy states, additional electronic states near the Fermi level were formed by introducing the superalkali cations. Such studies have the capability to crease the excitation diffusion length at a longer wavelength and promote hole mobility. Berry-phase simulations demonstrate considerable spontaneous polarization in both novel materials [Li_3_O]PbI_3_ and [Li_3_S]PbI_3_ having non-centrosymmetric structures (lacking inversion symmetry) and thus, allow this material to be used in communications, ferroelectrics, and remote sensing in a prolonged spectrum region.

Taking a quick glimpse at a different type of superalkali cation, a series of interesting computational experiments on the structural, stability/energetics, and electronic feature analyses of some new series of interesting non-metallic superalkali cations such as chain-shaped F_n_H_n+1_^+^ species, O_x_H_2x+1_^+^ clusters, and N_n_H_3n+1_^+^ species were reported by Srivastava (2019a), Srivastava (2019d), and Srivastava (2019c).

## Concluding remarks and future perspectives

The recent advances in the theoretical development, characterization, and first-time use of superalkalis are outlined in this review. In this report, we have made an effort to provide a general overview of modeling/design and characterization (theoretically) of superalkalis and their applications including sharing predictions for their future growth in the hopes of pointing designers in the right direction as they continue to create and use these special species. Efforts have been made to unveil the potential of some novel superalkali cations (Li_3_^+^) and C_6_Li_6_ type species for capturing and storing CO_2_/N_2_ molecules. The first-order hyperpolarizability-based NLO response properties of many new computationally designed superalkali-based materials (for example, fullerenes such as mixed-superalkali-doped B_12_N_12_ and B_12_P_12_ nanoclusters having good UV transparency and novel inorganic aromatic mixed-valent superalkali CaN_3_Ca as an alkali-earth-based high sensitivity multi-state NLO molecular switch, and novel lead-based halide perovskites formed by introducing the superalkali species) which can, indeed, be used as a new kind of electronic nanodevice for designing hi-tech NLO materials have been unraveled in this review. Understanding the mere interactions of alkalides in a solution, as well as in the gas phase where the potential to influence how such systems can be extended and applied in the future, is also highlighted in this survey. Understanding earned from this report will, indeed, assist in the discovery of novel superalkalis and will enrich the library’s arsenal of supersalts.

Although, based on this review, we can predict the forthcoming directions at the industrial level, there are still tasks to better understand the fascinating features of superalkalis and their characteristics to be measured precisely in the fields of inorganic, organic, and material chemistry. We are hopeful that further experiments, as well as theoretical investigations, will be capable of developing the present strategies and contributing to our future sights in better understanding the attention-grabbing features of a variety of already synthesized and designed/modeled novel superalkalis or related species. Moreover, the reported model complexes (as building new materials) in this report will be advantageous, and these may pave alternative pathways for experimentally rewarding applications and can have potential uses in advanced synthetic chemistry as catalysts.

## References

[B1] Anusiewicz I. (2010). The Na2X superalkali species (X=SH , SCH3 , OCH3 , CN , N3 ) as building blocks in the Na2XY salts ( Y = MgCl3 , Cl , NO2 ). An ab initio study of the electric properties of the Na2XY salts. Aust. J. Chem. 63, 1573–1581. 10.1071/CH10160

[B2] Awasthi S. Gaur J. K. Pandey S. K. Bobji M. S. Srivastava C. (2021a). High-strength , strongly bonded nanocomposite hydrogels for cartilage repair. ACS Appl. Mat. Interfaces 13, 24505–24523. 10.1021/acsami.1c05394 34027653

[B3] Awasthi S. Pandey S. K. Arunan E. Srivastava C. (2021b). A review on hydroxyapatite coatings for the biomedical applications: Experimental and theoretical perspectives. J. Mat. Chem. B 9, 228–249. 10.1039/d0tb02407d 33231240

[B4] Banjade H. R. DeepikaGiri S. Sinha S. Fang H. Jena P. (2021). Role of size and composition on the design of superalkalis. J. Phys. Chem. A 125, 5886–5894. 10.1021/acs.jpca.1c02817 34185533

[B5] Bano R. Ayub K. Mahmood T. Arshad M. Sharif A. Tabassum S. (2022). Mixed superalkalis are a better choice than pure superalkalis for B12N12 nanocages to design high-performance nonlinear optical materials. Dalton Trans. 51, 8437–8453. 10.1039/d2dt00321j 35593348

[B6] Barbatti M. Jalbert G. Antonio M. Nascimento C. (2002). Clustering of hydrogen molecules around a molecular Cation: the Li_3_ ^+^(H_2_)<sub>n</sub> clusters (n = 1 − 6). J. Phys. Chem. A 106, 551–555. 10.1021/jp013159m

[B7] Brédas J. L. Adant C. Tackx P. Persoons A. Pierce B. M. (1994). Third-order nonlinear optical response in organic materials: Theoretical and experimental aspects. Chem. Rev. 94, 243–278. 10.1021/cr00025a008

[B8] Chakraborty A. Giri S. Chattaraj P. K. (2010). Trapping of noble gases (He-Kr) by the aromatic H3+ and Li3+ species: A conceptual DFT approach. New J. Chem. 34, 1936–1945. 10.1039/c0nj00040j

[B9] Chemla D. S. Zyss J. (1987). Nonlinear optical properties of organic molecules and crystals. New York: Elsevier Inc. 10.1016/B978-0-12-170612-8.X5001-9

[B10] Czapla M. Skurski P. (2017). Oxidizing CO2 with superhalogens. Phys. Chem. Chem. Phys. 19, 5435–5440. 10.1039/c6cp08043j 28165083

[B11] Fernandez-Lima F. A. Vilela-Neto O. P. Pimentel A. S. Ponciano C. R. Caher-Nascimento M. A. Silveira E. F. d. (2009). Theoretical and experimental study of negative LiF clusters produced by fast ion impact on a polycrystalline ^7^LiF target. J. Phys. Chem. A 113, 15031–15040. 10.1021/jp905138d 19685887

[B12] Geskin V. M. Lambert C. Brédas J. L. (2003). Origin of high second- and third-order nonlinear optical response in ammonio/borato diphenylpolyene zwitterions: The remarkable role of polarized aromatic groups. J. Am. Chem. Soc. 125, 15651–15658. 10.1021/ja035862p 14664614

[B13] Giri S. Chakraborty A. Chattaraj P. K. (2011). Potential use of some metal clusters as hydrogen storage materials-a conceptual DFT approach. J. Mol. Model. 17, 777–784. 10.1007/s00894-010-0761-1 20556447

[B14] Giri S. Reddy G. N. Jena P. (2016). Organo-zintl clusters [P7R4]: A new class of superalkalis. J. Phys. Chem. Lett. 7, 800–805. 10.1021/acs.jpclett.5b02892 26882875

[B15] Gutsev G. L. Boldyrev A. I. (1982). DVM xα calculations on the electronic structure of “superalkali” cations. Chem. Phys. Lett. 92, 262–266. 10.1016/0009-2614(82)80272-8

[B16] Gutsev G. L. Boldyrev A. I. (1981). DVM-Xα calculations on the ionization potentials of MXk+1- complex anions and the electron affinities of MXk+1 “superhalogens. Chem. Phys. 56, 277–283. 10.1016/0301-0104(81)80150-4

[B17] Gutsev G. L. Boldyrev A. I. (1987). The electronic structure of superhalogens and superalkalies. Russ. Chem. Rev. 56, 519–531. 10.1070/RC1987v056n06ABEH003287

[B18] Gutsev G. L. Boldyrev A. I. (1985). The theoretical investigation of the electron affinity of chemical compounds. Adv. Chem. Phys. 61, 169–221. 10.1002/9780470142851.ch3

[B19] Honea E. C. Homer M. L. Labastie P. Whetten R. L. (1989). Localization of an excess electron in sodium halide clusters. Phys. Rev. Lett. 63, 394–397. 10.1103/physrevlett.63.394 10041062

[B20] Honea E. C. Homer M. L. Whetten R. L. (1993). Electron binding and stability of excess-electron alkali halide clusters: Localization and surface states. Phys. Rev. B 47, 7480–7493. 10.1103/PhysRevB.47.7480 10004742

[B21] Hou J. H. Wu D. Liu J. Y. Li S. Y. Yu D. Li Y. (2018). The effect of hydration on the electronic structure and stability of the superalkali cation Li3+. Phys. Chem. Chem. Phys. 20, 15174–15182. 10.1039/c8cp00862k 29789818

[B22] Hou N. Li Y. Wu D. Li Z. R. (2013). Do nonmetallic superalkali cations exist? Chem. Phys. Lett. 575, 32–35. 10.1016/j.cplett.2013.05.014

[B23] Jansen M. Z. (1977). Meue untersuchungen an Na3N03. Z. Anorg. Allg. Chem. 435, 13–20. 10.1002/zaac.19774350102

[B24] Khanna S. Jena P. (1995). Atomic clusters: Building blocks for a class of solids. Phys. Rev. B 51, 13705–13716. 10.1103/PhysRevB.51.13705 9978175

[B25] Khanna S. N. Jena P. (2011). Assembling crystals from clusters. Phys. Rev. Lett. 69, 1664–1667. 10.1103/PhysRevLett.69.1664 10046282

[B26] Koput J. (2008). *Ab initio* study on the structure and vibration-rotation energy levels of dilithium monofluoride. J. Chem. Phys. 129, 154306. 10.1063/1.2996108 19045192

[B27] Kudo H. Wu C. H. Ihle H. R. (1978). Mass-spectrometric study of the vaporization of Li2O(s) and thermochemistry of gaseous LiO, Li2O, Li3O, and Li2O2. J. Nucl. Mater. 78, 380–389. 10.1016/0022-3115(78)90460-9

[B28] Li Y. Wu D. Li Z. R. (2008). Compounds of superatom clusters: Preferred structures and significant nonlinear optical properties of the BLi6-X (X = F, LiF2, BeF3, BF4) motifs. Inorg. Chem. 47, 9773–9778. 10.1021/ic800184z 18831577

[B29] Li Y. Wu D. Li Z. R. Sun C. C. (2007). Structural and electronic properties of boron-doped lithium clusters: *Ab initio* and DFT studies. J. Comput. Chem. 28, 1677–1684. 10.1002/jcc.20637 17342718

[B30] Lias S. G. Bartmess J. E. Liebman J. F. Homes J. L. Levin R. D. Mallard W. G. (1988). Gas-phase ion and neutral thermochemistry. J. Phys. Chem. Ref. Data 17, 872.

[B31] Marder S. R. (2006). Organic nonlinear optical materials: Where we have been and where we are going. Chem. Commun. 2006, 131–134. 10.1039/b512646k 16372084

[B32] Nešković O. M. Veljković M. V. Veličković S. R. Petkovska L. T. Perić-Grujić A. A. (2003). Ionization energies of hypervalent Li2F, Li2Cl and Na2Cl molecules obtained by surface ionization electron impact neutralization mass spectrometry. Rapid Commun. Mass Spectrom. 17, 212–214. 10.1002/rcm.896 12539186

[B33] Omidvar A. (2018). Design of a novel series of donor-acceptor frameworks via superalkali-superhalogen assemblage to improve the nonlinear optical responses. Inorg. Chem. 57, 9335–9347. 10.1021/acs.inorgchem.8b01322 29995400

[B34] Otten A. Meloni G. (2018). Stability of lithium substituted silyls superalkali species. Chem. Phys. Lett. 692, 214–223. 10.1016/j.cplett.2017.12.044

[B35] Paduani C. Rappe A. M. (2017). Tuning the gap of lead-based halide perovskites by introducing superalkali species at the cationic sites of ABX3-type structure. Phys. Chem. Chem. Phys. 19, 20619–20626. 10.1039/c7cp02091k 28737790

[B36] Pal R. Poddar A. Chattaraj P. K. (2021). Atomic clusters: Structure, reactivity, bonding, and dynamics. Front. Chem. 9, 730548–730624. 10.3389/fchem.2021.730548 34485247 PMC8415529

[B37] Pan S. Contreras M. Romero J. Reyes A. Chattaraj P. K. Merino G. (2013a). C5Li7+ and O2Li 5+ as noble-gas-trapping agents. Chem. Eur. J. 19, 2322–2329. 10.1002/chem.201203245 23296901

[B38] Pan S. Jalife S. Romero J. Reyes A. Merino G. Chattaraj P. K. (2013b). Attractive Xe-Li interaction in Li-decorated clusters. Comput. Theor. Chem. 1021, 62–69. 10.1016/j.comptc.2013.06.026

[B39] Pan S. Merino G. Chattaraj P. K. (2012). The hydrogen trapping potential of some Li-doped star-like clusters and super-alkali systems. Phys. Chem. Chem. Phys. 14, 10345–10350. 10.1039/c2cp40794a 22735183

[B40] Pandey S. K. Arunan E. (2021). Effects of multiple OH/SH substitution on the H‐Bonding/Stability versus aromaticity of benzene rings: From computational insights. ChemistrySelect 6, 5120–5139. 10.1002/slct.202100689

[B41] Pandey S. K. (2021a). Computational study on the structure, stability, and electronic feature analyses of trapped halocarbons inside a novel bispyrazole organic molecular cage. ACS Omega 6, 11711–11728. 10.1021/acsomega.1c01019 34056325 PMC8154030

[B42] Pandey S. K. (2021b). Novel and polynuclear K- and Na-based superalkali hydroxides as superbases better than Li-related species and their enhanced properties: An ab initio exploration. ACS Omega 6, 31077–31092. 10.1021/acsomega.1c04395 34841150 PMC8613824

[B43] Parida R. Das S. Karas L. J. Wu J. I. C. Roymahapatra G. Giri S. (2019). Superalkali ligands as a building block for aromatic trinuclear Cu(i)-NHC complexes. Inorg. Chem. Front. 6, 3336–3344. 10.1039/c9qi00873j

[B44] Parida R. Reddy G. N. Ganguly A. Roymahapatra G. Chakraborty A. Giri S. (2018). On the making of aromatic organometallic superalkali complexes. Chem. Commun. 54, 3903–3906. 10.1039/c8cc01170b 29610800

[B45] Park H. Meloni G. (2017). Reduction of carbon dioxide with a superalkali. Dalton Trans. 46, 11942–11949. 10.1039/c7dt02331f 28853465

[B46] Prasad P. N. Williams D. J. (1991). Introduction to nonlinear optical effects in molecules and polymers. New York: John Wiley & Sons.

[B47] Reber A. C. Khanna S. N. Castleman A. W. (2007). Superatom compounds, clusters, and assemblies: Ultra alkali motifs and architectures. J. Am. Chem. Soc. 129, 10189–10194. 10.1021/ja071647n 17655299

[B48] Reber A. C. Khanna S. N. (2017). Superatoms: Electronic and geometric effects on reactivity. Acc. Chem. Res. 50, 255–263. 10.1021/acs.accounts.6b00464 28182404

[B49] Reddy G. N. Giri S. (2016). Organic heterocyclic molecules become superalkalis. Phys. Chem. Chem. Phys. 18, 24356–24360. 10.1039/c6cp04430a 27530344

[B50] Reddy G. N. Kumar A. V. Parida R. Chakraborty A. Giri S. (2018a). Zintl superalkalis as building blocks of supersalts. J. Mol. Model. 24, 306. 10.1007/s00894-018-3806-5 30291446

[B51] Reddy G. N. Parida R. Chakraborty A. Giri S. (2018b). Deltahedral organo-zintl superhalogens. Chem. Eur. J. 24, 13654–13658. 10.1002/chem.201802713 30011359

[B52] Rehm E. Schleyer P. v. R. Boldyrev A. I. (1992). *Ab initio* study of superalkalis. First ionization potentials and thermodynamic stability. Inorg. Chem. 31, 4834–4842. 10.1021/ic00049a022

[B53] Riedel R. Seel A. G. Malko D. Miller D. P. Sperling B. T. Choi H. (2021). Superalkali-alkalide interactions and ion pairing in low-polarity solvents. J. Am. Chem. Soc. 143, 3934–3943. 10.1021/jacs.1c00115 33660507 PMC8028040

[B54] Schleyer P. v. R. Würthwein E. U. Kaufmann E. Clark T. Pople J. A. (1983). Effectively hypervalent molecules. 2. Lithium carbide (CLi5), lithium carbide (CLi6), and the related effectively hypervalent first row molecules, CLi5-nHn and CLi6-nHn. J. Am. Chem. Soc. 105, 5930–5932. 10.1021/ja00356a045

[B55] Sinha S. Jena P. Giri S. (2022). Functionalized nona-silicide [Si9R3] zintl clusters: A new class of superhalogens. Phys. Chem. Chem. Phys. 24, 21105–21111. 10.1039/d2cp02619h 36018293

[B56] Srivastava A. K. (2019a). *Ab initio* investigations on non-metallic chain-shaped F H+1+ series of superalkali cations. Chem. Phys. Lett. 721, 7–11. 10.1016/j.cplett.2019.02.021

[B57] Srivastava A. K. (2019b). CO2-activation and enhanced capture by C6Li6: A density functional approach. Int. J. Quantum Chem. 119, 1–8. 10.1002/qua.25904

[B58] Srivastava A. K. (2019c). Design of the N: NH3 n+1+ series of “non-metallic” superalkali cations. New J. Chem. 43, 4959–4964. 10.1039/c8nj06126b

[B59] Srivastava A. K. Misra N. (2015). *Ab initio* investigations on the gas phase basicity and nonlinear optical properties of FLinOH species (n = 2-5). RSC Adv. 5, 74206–74211. 10.1039/c5ra14735b

[B60] Srivastava A. K. Misra N. (2016). OLi3O- anion: Designing the strongest base to date using OLi3 superalkali. Chem. Phys. Lett. 648, 152–155. 10.1016/j.cplett.2016.02.010

[B61] Srivastava A. K. (2019d). O H2+1+ clusters: A new series of non-metallic superalkali cations by trapping H3O+ into water. J. Mol. Graph. Model. 88, 292–298. 10.1016/j.jmgm.2019.02.010 30826709

[B62] Srivastava A. K. Pandey S. K. Misra N. (2016a). (CH3Br⋯NH3)@C60: The effect of nanoconfinement on halogen bonding. Chem. Phys. Lett. 662, 240–243. 10.1016/j.cplett.2016.09.036

[B63] Srivastava A. K. Pandey S. K. Misra N. (2016b). Prediction of superalkali@C60 endofullerenes, their enhanced stability and interesting properties. Chem. Phys. Lett. 655–656, 71–75. 10.1016/j.cplett.2016.05.039

[B64] Srivastava A. K. (2018). Single- and double-electron reductions of CO2 by using superalkalis: An *ab initio* study. Int. J. Quantum Chem. 118, 255988–e25606. 10.1002/qua.25598

[B65] Srivastava A. K. Srivastava H. Tiwari A. Misra N. (2022). X(CH3)+1+ superalkali cations (X = F, O and N) with methyl ligands. Chem. Phys. Lett. 790, 139352. 10.1016/j.cplett.2022.139352

[B66] Srivastava H. Srivastava A. K. (2022). Superalkalis for the activation of carbon dioxide: A review. Front. Phys. 10, 870205. 10.3389/fphy.2022.870205

[B67] Sun W. M. Cheng X. Ye Y. L. Li X. H. Ni B. L. (2022). On the possibility of using aza-cryptands to design superalkalis. Organometallics 41, 412–417. 10.1021/acs.organomet.1c00674

[B68] Sun W. M. Fan L. T. Li Y. Liu J. Y. Wu D. Li Z. R. (2014a). On the potential application of superalkali clusters in designing novel alkalides with large nonlinear optical properties. Inorg. Chem. 53, 6170–6178. 10.1021/ic500655s 24874698

[B69] Sun W. M. Li Y. Li X. H. Wu D. He H. M. Li C. Y. (2016a). Stability and nonlinear optical response of alkalides that contain a completely encapsulated superalkali cluster. ChemPhysChem 17, 2672–2678. 10.1002/cphc.201600389 27219640

[B70] Sun W. M. Li Y. Wu D. Li Z. R. (2013). Designing aromatic superatoms. J. Phys. Chem. C 117, 24618–24624. 10.1021/jp408810e

[B71] Sun W. M. Wu D. Kang J. Li C. Y. Chen J. H. Li Y. (2018). Decorating zintl polyanions with alkali metal cations: A novel strategy to design superatom cations with low electron affinity. J. Alloys Compd. 740, 400–405. 10.1016/j.jallcom.2017.12.075

[B72] Sun W. M. Wu D. Li X. H. Li Y. Chen J. H. Li C. Y. (2016b). Quasi-chalcogen characteristics of Al12Be: A new member of the three-dimensional periodic table. J. Phys. Chem. C 120, 2464–2471. 10.1021/acs.jpcc.5b11917

[B73] Sun W. M. Wu D. Li Y. Li Z. R. (2014b). Theoretical study on superalkali (Li3) in ammonia: Novel alkalides with considerably large first hyperpolarizabilities. Dalton Trans. 43, 486–494. 10.1039/c3dt51559a 24121411

[B74] Sun W. M. Wu D. (2019). Recent progress on the design, characterization, and application of superalkalis. Chem. Eur. J. 25, 9568–9579. 10.1002/chem.201901460 31025432

[B75] Sun W. M. Zhang X. L. Pan K. Y. Chen J. H. Wu D. Li C. Y. (2019). On the possibility of using the jellium model as a guide to design bimetallic superalkali cations. Chem. Eur. J. 25, 4358–4366. 10.1002/chem.201806194 30681743

[B76] Tkachenko N. V. Sun Z. M. Boldyrev A. I. (2019). Record low ionization potentials of alkali metal complexes with crown ethers and cryptands. ChemPhysChem 20, 2060–2062. 10.1002/cphc.201900422 31184431

[B77] Tong J. Li Y. Wu D. Li Z. R. Huang X. R. (2011). *Ab initio* investigation on a new class of binuclear superalkali cations M_2_Li_2<i>k</i>+1_ ^+^ (F_2_Li_3_ ^+^, O_2_Li_5_ ^+^, N_2_Li_7_ ^+^, and C_2_Li_9_ ^+^). J. Phys. Chem. A 115, 2041–2046. 10.1021/jp110417z 21332234

[B78] Tong J. Li Y. Wu D. Li Z. R. Huang X. R. (2009). Low ionization potentials of binuclear superalkali B2Li 11. J. Chem. Phys. 131, 164307. 10.1063/1.3254835 19894947

[B79] Tong J. Li Y. Wu D. Wu Z. J. (2012). Theoretical study on polynuclear superalkali cations with various functional groups as the central core. Inorg. Chem. 51, 6081–6088. 10.1021/ic202675j 22587710

[B80] Tong J. Wu Z. Li Y. Wu D. (2013). Prediction and characterization of novel polynuclear superalkali cations. Dalton Trans. 42, 577–584. 10.1039/c2dt31429k 23104219

[B81] Tu C. Yu G. Yang G. Zhao X. Chen W. Li S. (2014). Constructing (super)alkali-boron-heterofullerene dyads: An effective approach to achieve large first hyperpolarizabilities and high stabilities in M3O-BC59 (M = Li, Na and K) and K@n-BC59 (n = 5 and 6). Phys. Chem. Chem. Phys. 16, 1597–1606. 10.1039/c3cp53639d 24317581

[B82] Ullah F. Kosar N. Ayub K. Gilani M. A. Mahmood T. (2019). Theoretical study on a boron phosphide nanocage doped with superalkalis: Novel electrides having significant nonlinear optical response. New J. Chem. 43, 5727–5736. 10.1039/C9NJ00225A

[B83] Velickovic S. Djordjevic V. Cveticanin J. Djustebek J. Veljkovic M. Neskovic O. (2006). Ionization energies of LinX(n=2, 3; X=Cl, Br, I) molecules. Rapid Commun. Mass Spectrom. 20, 3151–3153. 10.1002/rcm.2712 16986212

[B84] Veličković S. R. Djustebek J. B. Veljković F. M. Radak B. B. Veljković M. V. (2012). Formation and ionization energies of small chlorine-doped lithium clusters by thermal ionization mass spectrometry. Rapid Commun. Mass Spectrom. 26, 443–448. 10.1002/rcm.6122 22279020

[B85] Veličković S. R. Koteski V. J. Čavor J. N. B. Djordjević V. R. Cvetićanin J. M. Djustebek J. B. (2007). Experimental and theoretical investigation of new hypervalent molecules LinF (n = 2-4). Chem. Phys. Lett. 448, 151–155. 10.1016/j.cplett.2007.09.082

[B86] Veličković S. R. Veljković F. M. Perić-Grujić A. A. Radak B. B. Veljković M. V. (2011). Ionization energies of K2X (X=F, Cl, Br, I) clusters. Rapid Commun. Mass Spectrom. 25, 2327–2332. 10.1002/rcm.5128 21766375

[B87] Wang B. Q. Li Z. R. Wu D. Wang F. F. (2007). Structures and static electric properties of novel alkalide anions F -Li+Li- and F-Li3+Li3-. J. Phys. Chem. A 111, 6378–6382. 10.1021/jp071218b 17580832

[B88] Wang D. Graham J. D. Buytendyk A. M. Bowen K. H. (2011). Photoelectron spectroscopy of the molecular anions, Li3O - and Na3O-. J. Chem. Phys. 135, 164308. 10.1063/1.3657854 22047240

[B89] Wang J. J. Zhou Z. J. Bai Y. Liu Z. B. Li Y. Wu D. (2012). The interaction between superalkalis (M3O, M = Na, K) and a C20F20 cage forming superalkali electride salt molecules with excess electrons inside the C20F20 cage: Dramatic superalkali effect on the nonlinear optical property. J. Mat. Chem. 22, 9652–9657. 10.1039/c2jm15405f

[B90] Wang X. B. Ding C. F. Wang L. S. Boldyrev A. I. Simons J. (1999). First experimental photoelectron spectra of superhalogens and their theoretical interpretations. J. Chem. Phys. 110, 4763–4771. 10.1063/1.478386

[B91] Wang Y. F. Qin T. Tang J. M. Liu Y. J. Xie M. Li J. (2020). Novel inorganic aromatic mixed-valent superalkali electride CaN3Ca: An alkaline-earth-based high-sensitivity multi-state nonlinear optical molecular switch. Phys. Chem. Chem. Phys. 22, 5985–5994. 10.1039/c9cp06848a 32123888

[B92] Wei T. Dahiya H. Liang X. Zhu W. Pandey S. K. Singh M. K. (2022). Bulk heterojunction organic photovoltaic cells based on D–A type BODIPY small molecules as non-fullerene acceptors. J. Mat. Chem. C Mat. 10, 12776–12788. 10.1039/d2tc02497g

[B93] Wright K. W. A. Rogers D. E. Lane I. C. (2009). Geometric bonding effects in the X [sup 2]A[sub 1], A [sup 2]Σ[sub u] [sup +], and B [sup 2]Π[sub g] states of Li[sub 2]F. J. Chem. Phys. 131, 104306. 10.1063/1.3216373

[B94] Wu C. H. Kudo H. Ihle H. R. (1979). Thermochemical properties of gaseous Li3O and Li2O2. J. Chem. Phys. 70, 1815–1820. 10.1063/1.437656

[B95] Xu H. L. Li Z. R. Wu D. Wang B. Q. Li Y. Gu F. L. (2007). Structures and large NLO responses of new electrides: Li-Doped fluorocarbon chain. J. Am. Chem. Soc. 129, 2967–2970. 10.1021/ja068038k 17305338

[B96] Yang H. Li Y. Wu D. Li Z. R. (2012). Structural properties and nonlinear optical responses of superatom compounds BF4-M (M = Li, FLi2, OLi3, NLi4). Int. J. Quantum Chem. 112, 770–778. 10.1002/qua.23053

[B97] Yang H. Wu D. He H. M. Yu D. Li Y. Li Z. R. (2018). The behavior of the aluminum trimer when combining with different superatom clusters. RSC Adv. 8, 6667–6674. 10.1039/c7ra12852e 35540389 PMC9078306

[B98] Ye Y. L. Pan K. Y. Ni B. L. Sun W. M. (2022). Designing special nonmetallic superalkalis based on a cage-like adamanzane complexant. Front. Chem. 10, 853160–853167. 10.3389/fchem.2022.853160 35360533 PMC8963935

[B99] Yi X. G. Wang Y. F. Zhang H. R. Cai J. H. Liu X. X. Li J. (2022). Can a molecular switch exist in both superalkali electride and superalkalide forms? Phys. Chem. Chem. Phys. 24, 5690–5699. 10.1039/d1cp05657c 35187550

[B100] Yokoyama K. Haketa N. Tanaka H. Furukawa K. Kudo H. (2000). Ionization energies of hyperlithiated Li2F molecule and Li F−1 (n=3, 4) clusters. Chem. Phys. Lett. 330, 339–346. 10.1016/S0009-2614(00)01109-X

[B101] Yu D. Wu D. Liu J. Y. Li Y. Sun W. M. (2020). Unveiling the potential of superalkali cation Li3+for capturing nitrogen. Phys. Chem. Chem. Phys. 22, 26536–26543. 10.1039/d0cp03769a 33188670

[B102] Zein S. Ortiz J. V. (2011). Interpretation of the photoelectron spectra of superalkali species: Li 3O and Li3O-. J. Chem. Phys. 135, 164307. 10.1063/1.3636082 22047239

[B103] Zhang X. L. Ye Y. L. Zhang L. Li X. H. Yu D. Chen J. H. (2021a). Designing an alkali-metal-like superatom Ca3B for ambient nitrogen reduction to ammonia. Phys. Chem. Chem. Phys. 23, 18908–18915. 10.1039/d1cp01533h 34612429

[B104] Zhang X. L. Zhang L. Ye Y. L. Li X. H. Ni B. L. Li Y. (2021b). On the role of alkali-metal-like superatom Al12P in reduction and conversion of carbon dioxide. Chem. Eur. J. 27, 1039–1045. 10.1002/chem.202003733 32969553

[B105] Zhang Z. Chen H. (2019). Superalkali NM4 (M = Li, Na, K): Stabilities and electronic structures. Phys. Lett. A 383, 125952. 10.1016/j.physleta.2019.125952

[B106] Zintl E. Morawietz W. (1938). Orthosalze von Sauerstoffsäuren. Z. Anorg. Allg. Chem. 236, 372–410. 10.1002/zaac.19382360134

